# *Allophylastrum*: a new genus of Sapindaceae from northern South America

**DOI:** 10.3897/phytokeys.5.1684

**Published:** 2011-07-28

**Authors:** Pedro Acevedo-Rodríguez

**Affiliations:** Department of Botany, MRC-166 Smithsonian Institution, P.O. Box 37012, Washington D.C. 20013-7012, USA

**Keywords:** *Allophylastrum*, *Allophylus*, Sapindaceae, Roraima, Brazil, Guyana

## Abstract

The new genus *Allophylastrum* (Sapindaceae) is described from Roraima, Brazil and Guyana. *Allophylastrum* resembles the genus *Allophylus* in its vegetative morphology but differs by its apetalous flowers with a cupular nectary disc, 5–6 unequal stamens, and 4- to 5- porate pollen grains. A key is provided to differentiate *Allophylastrum* from *Allophylus*. The new species *Allophylastrum frutescens* is described and illustrated.

## Introduction

While working on a treatment of Sapindaceae for the Flora of the Guianas Project (Acevedo-Rodríguez in prep.), I came across a new species that although Sapindaceous in appearance, did not fit any of the known genera of Sapindaceae. A second collection of the new species from the adjacent Brazilian state of Roraima, was discovered later at Kew Gardens and New York Botanical Garden herbaria. The new genus resembles *Allophylus* because of its vegetative morphology, but its flowers and inflorescences definitely do not belong with it. Examination of pollen grains and DNA sequences (Wurdack et al. in prep.) indeed confirm the new genus to belong in Sapindaceae. However, because its flowers and inflorescences are so distinctive from *Allophylus*, a new genus is here proposed to accommodate the new species. *Allophylastrum* is preliminarily placed sister to *Allophylus* in the Paullinieae tribe (sensu Acevedo-Rodríguez et al. 2011) awaiting results from the analyses of additional genera of Sapindaceae.

## Taxonomic treatment

### Allophylastrum

Acev.-Rodr. gen. nov.

urn:lsid:ipni.org:names:77112773-1

http://species-id.net/wiki/Allophylastrum

#### Latin.

A Allophylus flore solitario, apetalo, nectaris cupulato differt

#### Type.

A. *frutescens* Acev.-Rodr.

#### Description.

Small trees or shrubs. Stipules wanting. Leaves alternate, trifoliolate; leaflets serrate. Flowers solitary, axillary or in short racemes, actinomorphic, unisexual with staminate flowers sometimes bearing a rudimentary 2-locular gynoecium; pedicels elongated, non-articulate; calyx 4-merous, sepals of similar length, in two whorls; petals wanting; disc cupular; stamens (5)6, the filaments slightly of unequal length, connate at base, ovary 2-locular, with a single ovule per locule. Fruit of 1–2 basally connate, indehiscent monocarps, with fleshy exocarp, and a semi-woody endocarp; seeds exarillate, with papery testa.

#### Distribution.

One species, known only from Guyana and Brazil (Roraima).

**Discusssion.** The new genus is morphologically similar to *Allophylus* as they share similar shrubby-arboreal habit, and trifoliolate leaves without stipules (Acevedo-Rodríguez et al. 2011).

#### Key to Allophylastrum and Allophylus

**Table:** 

1	Flowers actinomorphic, apetalous, solitary, or in short axillary racemes; nectary disc cupular; pedicel non-articulate, >4 times as long as the calyx; pollen 4- to 5-porate , 4- or 5-angled in polar view	*Allophylastrum*
–	Flowers zygomorphic, petaliferous, on lateral cincinni in a racemiform or paniculate thyrse; nectary disc unilateral, semi-annular, 2- to 4-lobed, or rarely annular; pedicels articulate, 1–2 times as long as the sepals; pollen 3-porate, triangular in polar view	*Allophylus*

#### Etymology.

The name *Allophylastrum* is proposed to indicate its close resemblance to *Allophylus*.

### Allophylastrum frutescens

Acev.-Rodr. sp. nov.

urn:lsid:ipni.org:names:77112774-1

http://species-id.net/wiki/Allophylastrum_frutescens

Fig. 1 

#### Latin.

Frutex vel arbor parva; folia trifoliolata; foliola chartacea, elliptica, margine serrato; flores apetali, monocarpus ellipsoideus, glabrus.

#### Type.

Brazil. Roraima, Sema Ecological Station, Ilha de Maracá, 3°22'N, 61°25'W, *E.S. Silva & J. Lima 5828* (holotype NY!, isotypes INPA, K!-2, U-2!).

#### Description.

Shrub or small tree to 7 m tall. Branches terete, glabrous, brown with grayish lineate lenticels. Leaves trifoliolate; petioles flattened-canaliculate adaxially, 1.6–4.5 cm long, minutely puberulent; leaflets elliptic, 6–10.8 × 1.3–3.4 cm (the lateral ones smaller), chartaceous, the base cuneate on distal leaflets, obtuse-acute and asymmetrical on lateral ones, the apex acute to acuminate, the margins serrate. Flowers axillary, solitary or in short (1–2 cm long), axillary racemes; pedicels 8–10 mm long; sepals 4, concave, obovate, rounded at apex, puberulent, ca. 2 mm long; disc cupular, glabrous, with sub-fimbriate margin; filaments glabrous, 4–5 mm long; pistillode ca. 0.4 mm long; pistillate flowers unknown. Monocarps ellipsoid, divaricate, glabrous, 1–1.2 cm long; exocarp red, fleshy and thin; endocarp subwoody. Seed obovoid, ca. 1 cm long, with papery testa.

**Figure 1.**
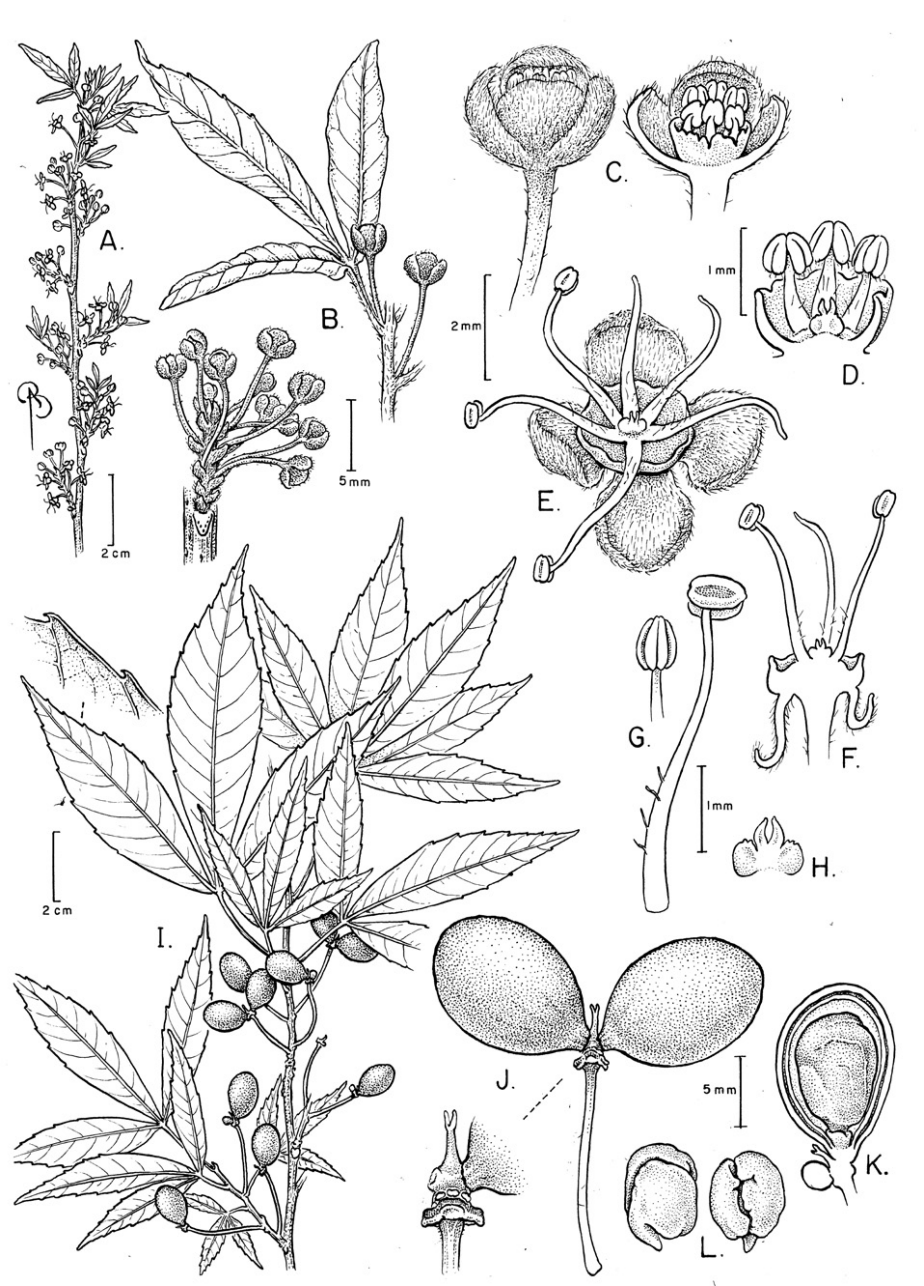
*Allophylastrum frutescens*. **A** Flowering branch **B** Detail of branch with leaf subtending a single flower, detail of a raceme **C** Pre-anthesis staminate flower and l.s. of same **D** Detail of staminate flower nectary disc, stamens, and pistillode **E** Staminate flower at anthesis, showing nectary disc and stamens **F** l.s. of staminate flower **G** Stamen, frontal and lateral views **H** Pistillode **I** Fruiting branch **J** Fruit with two monocarps and detail of monocarp insertion **K** Fruit showing fully developed monocarp with seed, and an undeveloped monocarp **L** Embryo, lateral and frontal views. **A–H** from *Silva and Lima 5828* (NY); **I–L** from *Lima 812* (NY).

#### Pollen.

**Figure 2.**
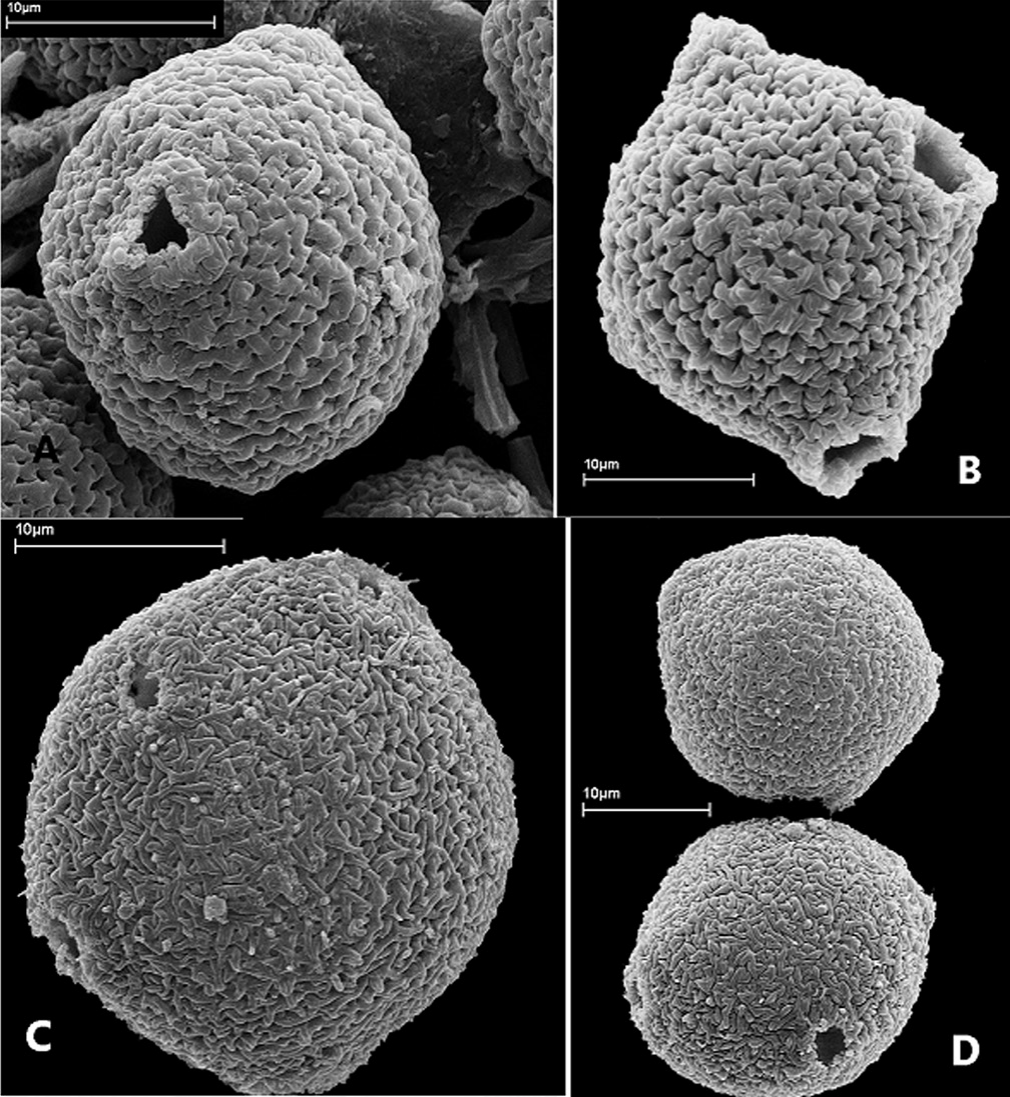
Pollen of *Allophylastrum frutescens*. **A** Equatorial view showing pore **B** Oblique polar view of 4-angular grain showing 2 pores **C** Oblique equatorial view of a 5-angular grain showing 3 pores **D** Polar view of 5-angualr grain (top), oblique equatorial views of a 5-angular grain showing 3 pores. All from *R.H. Schomburgk 336* (W).

Pollen grains in *Allophylastrum frutescens* are subglobose in equatorial view and obtusely 4- or 5-angled in polar view, 4–5-porate, with rugulate ornamentation (Fig. 2a-d). Size as measured from 20 pollen grains using light microscopy varies from 24.57–31.96 µm long by 21.86–28.10 µm wide. Generally, Sapindaceae pollen is 3-aperturate or less often 2- or 4-aperturate (Acevedo-Rodríguez et al. 2011). Therefore, this is the first time a 5-aperturate pollen is reported for the family. *Allophylastrum* pollen resembles that of *Allophylus* by being porate and having a rugulate ornamentation (Acevedo-Rodríguez et al. 2011); however the shape and the number of apertures is quite distinctive from that of *Allophylus*, where the pollen is triangular in polar view, oblate in equatorial view, and triporate.

#### Distribution and ecology.

Known only from Brazil (Roraima) and Guyana, on terra firme forest.

#### Specimens examined.

Guyana**:** Without locality, *M.R. Schomburgk 505* (B), *R.H. Schomburgk 336* (BM, K, NY, W). Brazil. Roraima, Mun. Boa Vista, road to Santa Rosa, secondary forest , *J. Lima 812* (INPA, K).

#### Note.

The above cited collections by *Schomburgk* were studied by L.A.T. Radlkofer (1829–1927) but wrongly identified as *Allophylus edulis* (St. Hil.) Hieron. ex Niederlein, a vegetatively similar species.

## Supplementary Material

XML Treatment for Allophylastrum

XML Treatment for Allophylastrum frutescens
